# Patient health questionnaire in the general population sample - establishing the cut-off score for detecting major depression

**DOI:** 10.1192/j.eurpsy.2023.225

**Published:** 2023-07-19

**Authors:** N. P. Maric, L. Mihic, C. Study Group

**Affiliations:** 1 Faculty of Medicine, University of Belgrade; 2 Institute of Mental Health, Belgrade; 3 Faculty of Philosophy, University of Novi Sad, Novi Sad; 4 Univerisity of Belgrade, Belgrade, Serbia

## Abstract

**Introduction:**

The traditional Patient Health Questionnaire (PHQ-9) cut-off score of ≥10 has been found to balance best sensitivity and specificity when used in patient populations. Depression screening has been recommended in general population surveys, however, in comparison to patient population a few studies have suggested different optimal cut-off values to detect possible depression.

**Objectives:**

Aim of this research involving country-representative general adult population sample was to identify which PHQ-9 cut-off score distinguishes individuals with and without depression.

**Methods:**

This was a cross-sectional observational epidemiological survey CoV2Soul.rs (registration number NCT04896983) using in-person interviews and multistage household probabilistic sampling in mid-2021 to recruit representative adult sample (N=1203; age 43.7 (SD 13.6); 48.7% male; mean education 12.7 (SD 2.9)). Current mental disorders were observer-rated on the Mini International Neuropsychiatric Interview (MINI Standard 7.0.2.). The PHQ-9 was self-rated by the participants and research assistants were not aware of their self-scoring. Sensitivity, specificity, and likelihood ratio tests for predicting current major depressive episode were evaluated at various cut-off points of the PHQ-9.

**Results:**

The mean PHQ-9 score was 3.2 (SD 3.8). The value is highly comparable with other general population studies. At the cut-off score of 8, sensitivity was .85 and specificity was .91. At the cut-off value of 10, sensitivity dropped to .74, suggesting that the optimal cut-off score was 8. ROC analysis showed that the area under the curve was .95, indicating that the Serbian PHQ-9 can discriminate very well between persons with and without depression (Figure 1).

**Table 1.** Sensitivity, specificity and likelihood ratio tests at various cut-off points of the PHQ-9

Cut-offs	Sensitivity	Specificity	LR+	LR-
≥ 5.5	.96	.82	5.33	0.05
≥ 6.5	.89	.87	6.73	0.13
≥ 7.5	.85	.91	9.06	0.16
≥ 8.5	.74	.94	11.95	0.28
≥ 9.5	.74	.96	16.84	0.27
≥ 10.5	.63	.96	17.03	0.39
≥ 11.5	.59	.97	21.18	0.42
≥ 12.5	.59	.98	31.21	0.42

**Conclusions:**

PHQ-9 is a highly useful screening tool, but the same cut-off score might not be appropriate in all settings. In European countries, studies of the general population that determine optimal cut-off PHQ-9 value against a validated interview to detect depression are rare. We demonstrated that the cut-off of ≥8 balances best its sensitivity and specificity when assessed against the structured diagnostic interview in the general population.

This work was supported by the Science Fund of the Republic of Serbia, grant number #7528289. The special research program on Covid-19 is financed by a World Bank loan through Serbia Accelerating Innovation and Entrepreneurship Project – SAIGE.

**Disclosure of Interest:**

None Declared

